# Dexmedetomidine vs Dexamethasone as an Adjuvant to Levobupivacaine in Ultrasound-Guided Transversus Abdominis Plane Block for Postoperative Analgesia in Patients Undergoing Total Abdominal Hysterectomies

**DOI:** 10.5812/aapm-142059

**Published:** 2023-12-10

**Authors:** Jyoti Sinha, Abhimanyu Singh Pokhriyal, Veena Asthana, Ruchira Nautiyal

**Affiliations:** 1 Department of Anesthesiology, Himalayan Institute of Medical Sciences, Jollygrant, Dehradun; 2 Department of Gynecology, Himalayan Institute of Medical Sciences, Jollygrant, Dehradun

**Keywords:** Hysterectomy, Dexamethasone, Dexmedetomidine, Levobupivacaine, TAP Block

## Abstract

**Background:**

In the postoperative period, open total abdominal hysterectomy (TAH) surgeries induce considerable pain. Multimodal strategies are being used to alleviate pain.

**Objectives:**

This study aimed to examine the efficacy and safety of dexamethasone and dexmedetomidine as an adjuvant to levobupivacaine in ultrasound-guided transversus abdominis plane (TAP) blocks for postoperative pain in TAH patients.

**Methods:**

A total of 72 patients with ASA grade I and grade II were randomly and equally assigned to two groups. After the completion of surgery with a subarachnoid block (SAB), patients in group 1 received a mixture of 20 mL of 0.25% levobupivacaine and 4 mg of dexamethasone on each side of the TAP block. Patients in group 2 received a mixture of 20 mL of 0.25% levobupivacaine and dexmedetomidine, with a total dose of 1 µg/kg body weight evenly distributed bilaterally in the TAP block. Patients were evaluated for pain using the Visual Analog Scale (VAS), total tramadol consumption as rescue analgesia, time to first rescue analgesia, any adverse effects, and patient satisfaction.

**Results:**

When comparing VAS scores for pain assessment, we observed that the mean VAS score was initially comparable between the two groups for the first hour. However, at 6, 9, and 12 h, VAS scores were significantly lower in group 2. The mean total tramadol consumption was higher in group 1 than in group 2 (213.33 ± 44.08 vs 161.11 ± 37.93 mg, P-value 0.027). The time to the first rescue analgesia after the TAP block in the postoperative period was significantly longer in group 2 (47.5 ± 62.76 vs 77.22 ± 56.14 min, P-value 0.002). No significant side effects were noted, and a greater proportion of patients in group 2 expressed satisfaction with their overall pain treatment.

**Conclusions:**

The addition of dexmedetomidine to levobupivacaine is superior to the addition of dexamethasone, as it prolongs the duration of the block in the dexmedetomidine group. However, the use of dexamethasone as an adjuvant is a good alternative option, particularly due to its lower cost and reduced incidence of adverse effects such as postoperative nausea and vomiting.

## 1. Background

Pain following total abdominal hysterectomy (TAH) can arise from various reasons, including incisional pain, pain from deeper (visceral) structures, and dynamic pain, such as during coughing or mobilization. However, a significant portion of postoperative pain is typically attributed to the abdominal wall incision (1).

To effectively reduce pain after TAH, a multimodal approach is essential, as unmanaged pain can lead to prolonged hospital stays (2). Opioids have traditionally been included in multimodal pain management strategies, but they come with the risk of severe side effects like drowsiness, nausea, vomiting, and respiratory depression, which can hinder early patient mobilization (1).

In this context, the transversus abdominis plane (TAP) block appears to be an ideal approach for alleviating postoperative pain in patients undergoing lower abdominal gynecological operations, especially when performed as part of a multimodal analgesic regimen (3). Between the TA and internal oblique (IO) muscles, there is a gap called TAP. In this procedure, a local anesthetic (LA) is injected into the area that may serve as the “TAP plane,” where the T6 to L1 nerve roots will be severed (4).

Adjuvants to local anesthetics, such as opioids, ketamine, dexamethasone, and alpha-2 agonists (such as dexmedetomidine), have been successfully used in peripheral nerve blocks and field blocks to extend the duration of postoperative analgesia (5).

Levobupivacaine, a local anesthetic agent, boasts a longer duration of action, greater safety, and lower toxic profile when compared to bupivacaine (6). Despite an extensive review of the literature, no studies comparing dexamethasone and dexmedetomidine as adjuvants to levobupivacaine in TAP blocks were found. However, there are conflicting results regarding the overall efficacy of these 2 additives as adjuvants in LA (7-9). Therefore, we conducted our study using levobupivacaine as a local anesthetic and dexamethasone or dexmedetomidine as an adjuvant in TAP blocks.

Dexmedetomidine has sedative, analgesic, and perioperative sympatholytic effects (10), while dexamethasone reduces pain by reducing inflammation and inhibiting pain-causing unmyelinated “C” fiber transmission (11).

The aim of our study was to assess the effectiveness of dexamethasone and dexmedetomidine as local anesthetic adjuvants to levobupivacaine in ultrasound-guided TAP blocks for comprehensive pain management in patients who have undergone open abdominal hysterectomies under SAB.

## 2. Objectives

This study aimed to assess the efficacy and safety of dexamethasone and dexmedetomidine as adjuvants to levobupivacaine in ultrasound-guided TAP blocks for patients undergoing total abdominal hysterectomies, with a focus on analgesia duration, analgesia quality, postoperative rescue analgesic consumption, and adverse effects or complications.

## 3. Methods

**Figure 1.**
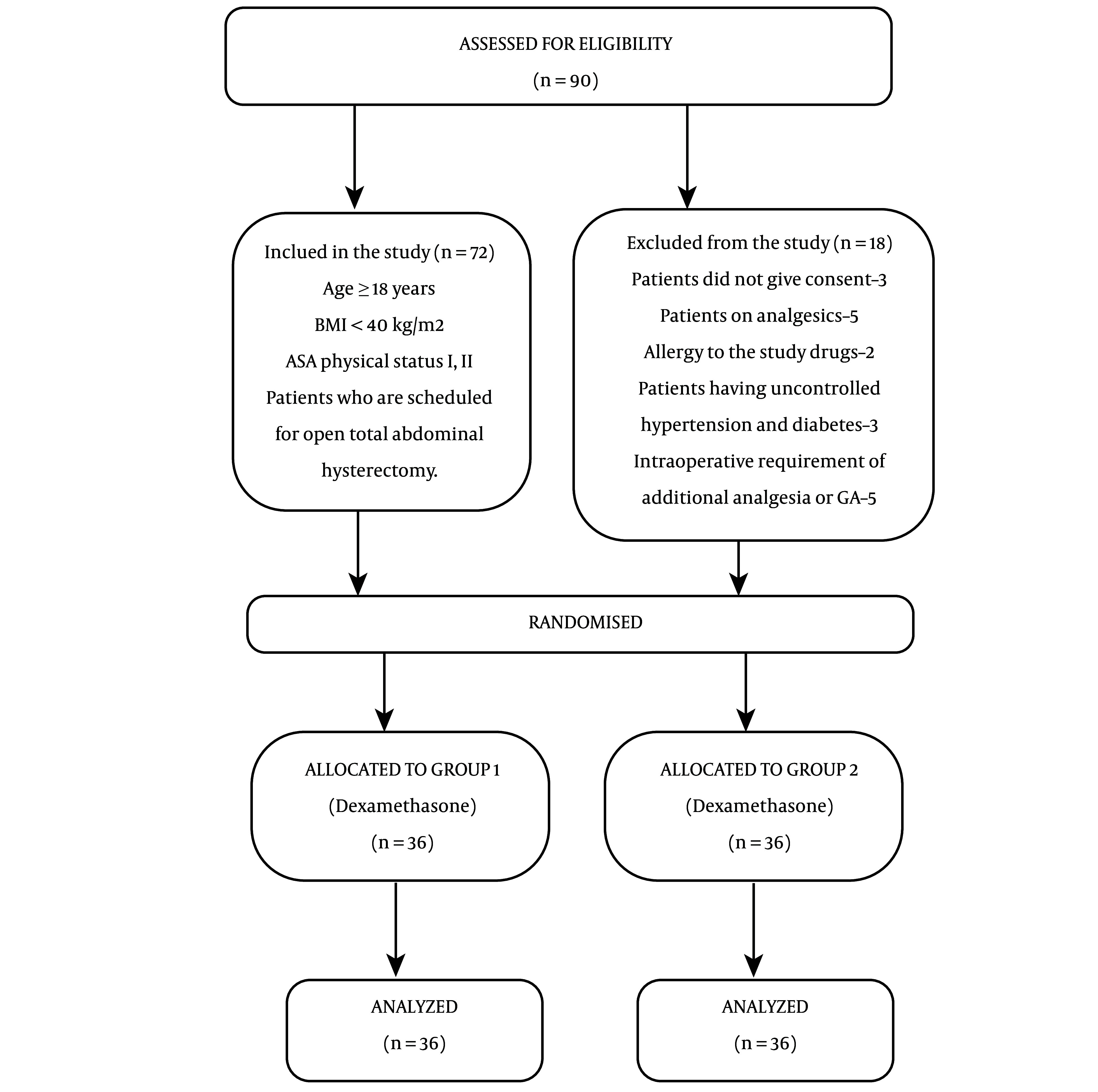
Consort flow diagram

The study was performed after obtaining written informed consent from the participants who were scheduled to undergo open TAH. The study received approval from the regional ethical council and scientific institutional review board and was registered in the Clinical Trials Registry of India (CTRI/2021/01/030607). Over the course of a year, the study was performed in a teaching hospital and tertiary care center. A total of 72 patients, classified as ASA grade I and grade II, aged ≥ 18 years, and scheduled for open TAH, were included in the study. Patients with a body mass index (BMI) > 40, chronic pain, bleeding/clotting disorders, hypersensitivity to the drugs used, uncontrolled hypertension, hepatic insufficiency (liver enzymes elevated more than 2 times the normal values), renal insufficiency (serum creatinine elevated more than 2 times the normal values), localized infection at the injection site, and those who refused to participate were excluded (Figure 1). 

All surgical candidates were instructed to abstain from solid food for 6 h and clear liquids for 2 h before the procedure. The night before and 2 h prior to surgery, patients received 0.25 mg of oral alprazolam and 150 mg of oral ranitidine. They were also provided with information about the procedure and instructed on how to use the Visual Analog Scale (VAS). In the operating room, patients were monitored for noninvasive blood pressure (BP), heart rate (HR), electrocardiography, and oxygen saturation (SpO_2_) after the placement of an 18G IV cannula. The anesthesia protocol was standardized for all patients. After confirming the L3 and L4 intervertebral space, a 25-gauge spinal needle was used to puncture the space in a midline approach, and 15 mg of 0.5% hyperbaric bupivacaine was administered after confirming the free flow of cerebro-spinal fluid (CSF). Surgery started after confirming an adequate level (T6). Patients requiring supplemental analgesia or general anesthesia (GA) during surgery were excluded from the study.

After surgery and as the subarachnoid block (SAB) sensory level regressed to the T10 dermatome, a TAP block was performed using ultrasonography after the closure of the skin. The patients were randomly divided into 2 groups: Odd and even. The even-numbered patients were assigned to receive levobupivacaine plus dexamethasone (group 1), while the odd-numbered patients were assigned to receive levobupivacaine plus dexmedetomidine (group 2). A linear array ultrasound probe (M TURBO, FUJIFILM Sonosite, Inc, 21919 Bothell, WA 98021, USA) was positioned at the level of the umbilicus on the anterolateral abdominal wall, between the subcostal edge and the iliac crest. This probe has a high frequency (6 - 13 MHz) and short wavelength (L4-12). The TA, IO, and external oblique muscles were all recognized. The IO and TA muscles form a hypoechoic line that points to the fascial plane. Under ultrasound guidance and after confirming a negative aspiration result, a local anesthetic solution combination was administered using a short-beveled 23-gauge spinal needle. In group 1, a local anesthetic solution consisting of 20 mL of 0.25% levobupivacaine and 4 mg of dexamethasone was given equally on both sides. In group 2 (on each side), patients received a mixture of 20 mL of 0.25% levobupivacaine and dexmedetomidine, with a total dose of 1 µg/kg body weight, evenly distributed bilaterally in the TAP block. Patients were then transferred to a post-anesthesia care unit where their HR, BP, and SpO_2_ were monitored. Pain levels were assessed using VAS at 1st, 3rd, 6th, 12th, 18th, and 24th h after the ultrasound-guided TAP block, starting from the 20th minute after arrival in the post-anesthesia care unit. Patients were asked to rate their postoperative pain on a 10-cm VAS scale, ranging from no pain (0) to very severe pain (10).

As the initial rescue analgesic, 1 g of intravenous paracetamol was administered. A tramadol patient-controlled anesthesia (PCA) pump was used as an additional analgesic for rescue analgesia. In the PCA pump, a tramadol concentration of 4 mg/mL was achieved, which was then adjusted to a 20 mg demand dose with a 10-min lockout interval and a 4-h limit of 100 mg.

Over the first 24 h after surgery, we evaluated the duration and quality of analgesia, postoperative analgesic consumption, and the presence of any adverse effects or complications.

The primary outcome was to measure the time to the first rescue analgesia. The secondary outcome was to measure total tramadol consumption over 24 h, VAS scores at different time intervals, patient satisfaction on postoperative day 1 using a Likert scale questionnaire, and any drug-related side effects or complications.

### 3.1. Statistical Analysis

Interventional, prospective, randomized, and comparative types of study

Sample size and selection procedures: The sample size for the study was calculated by the following formula:


$$n\;=\;\frac{(\sigma_{1}^{2}\;+\;\sigma_{2}^{2}).\;{\left[Z_{1-\;\alpha /2\;}\;+\;Z_{1-\;\beta }\;\right]}^{2}}{{\left(M_{1}\;-\;M_{2}\right)}^{2}}$$

Where n is the required sample size, Z_1- α/2 _is the critical value of the normal distribution at α/2, Z_1-β _is the critical value of the normal distribution at β, σ_1_, and σ_2_ are the SDs of the 2 groups, M_1_ and M_2_ are the means of the 2 groups. The critical value is 1.96 for a 95% CI, and α is 0.05. For a power of 90%, β is 0.1, and its critical value is 1.282.

Thus, a sample size of 36 patients per group was considered necessary to detect statistical significance with an effect size of 1.0 at alpha 0.05 and a power of 90%. We increased recruitment by more than 20% to compensate for unexpected losses.

Statistical analysis was performed using SPSS version 17.0. If the data were not uniformly distributed, continuous variables were reported as mean ± SD or median. Frequencies and percentages were used to express categorical variables. The Student’s *t*-test was used to compare the continuous variables between the groups. The chi-square test or Fisher’s exact test was used to compare nominal categorical data between the groups. The Mann-Whitney U-test was used to compare continuous variables with non-normal distributions. A P-value is a measure of probability, and a value less than 0.05 was considered significant in all statistical tests of our study.

## 4. Results

A total of 72 patients completed this study, and all patients followed the study protocol. Demographic variables such as age, weight, height, BMI, the duration of the procedure, and the duration of the sensory block due to spinal anesthesia in the 2 groups were comparable and showed no statistically significant difference (Table 1). 

**Table 1.** Comparison of Demographic Variables, Duration of Surgery, and Anesthesia Between Group 1 and Group 2 ^a^

Variables Name	Group 1	Group 2	P-Value
**Age**	45.56 ± 6.53	47.53 ± 8.06	0.258
**Height**	159.31 ± 7.32	159.11 ± 9.05	0.921
**Weight**	58.47 ± 5.04	59.64 ± 6.16	0.191
**BMI**	25.13 ± 3.09	23.60 ± 2.14	0.657
**Duration of sensory SAB**	160.83 ± 11.56	155.83 ± 14.42	0.109
**Duration of surgery**	121.94 ± 13.69	116.81 ± 13.64	0.107

Abbreviations: BMI, body mass index; SAB, subarachnoid block.

^a^ Results are expressed as mean ± SD.

The difference in mean VAS at 20 min and 1 h was found to be statistically insignificant in groups 1 and 2, respectively (mean ± SD: 1.25 ± 1.54, 1.36 ± 2.21 vs 1.19 ± 1.40, 1.21 ± 2, P-value > 0.05). However, there was a statistically significant difference between VAS scores at 6, 9, and 12 h (group 1: 3.06 ± 1.96, 2.39 ± 1.93, 1.67 ± 1.59 vs group 2: 1.72 ± 1.61, 0.92 ± 1.52, 0.69 ± 1.24; P-value 0.004, P-value 0.002 and P-value 0.001), and, at 18 and 24 h, the mean VAS score was comparable between the 2 groups (Table 2). 

**Table 2.** Comparison of Visual Analogue Scale Score at Different Time Intervals Between the 2 Groups ^a^

Variables Name	Group 1	Group 2	P-Value
**VAS Score_20 min**	1.25 ± 1.54	1.19 ± 1.40	0.439
**VAS Score_1 h**	1.36 ± 2.21	1.21 ± 2	0.396
**VAS Score_6 h**	3.06 ± 1.96	1.72 ± 1.61	0.004
**VAS Score_9 h**	2.39 ± 1.93	0.92 ± 1.52	0.001
**VAS Score_12 h**	1.67 ± 1.59	0.69 ± 1.24	0.001
**VAS Score_18 h**	1.53 ± 1.46	1.42 ± 1.61	0.797
**VAS Score_24 h**	1.81 ± 1.14	1.78 ± 1.66	0.466

Abbreviation: VAS, Visual Analogue Scale.

^a^ Results are expressed as mean ± SD.

Significant hemodynamic variability in terms of mean BP (Table 3) and mean HR (Table 4) was found between the groups at 1, 12, and 18 h after TAP block (P-value < 0.05).

**Table 3.** Comparison of Mean Blood Pressure at Different Time Intervals Between the 2 Groups ^a^

Variables Name	Group 1	Group 2	P-Value
**Mean BP_20 min**	89.25 ± 12.16	84.14 ± 9.93	0.055
**Mean BP_1 h**	91.61 ± 10.12	83.42 ± 9.18	0.001
**Mean BP_6 h**	89.47 ± 7.96	85.03 ± 9.18	0.0031
**Mean BP_9 h**	89.08 ± 8.72	83.64 ± 10.24	0.018
**Mean BP_12 h**	89.92 ± 9.08	83.56 ± 9.03	0.004
**Mean BP_18 h**	89.86 ± 9.99	83.58 ± 8.64	0.006
**Mean BP_24 h**	88.47 ± 8.96	83.94 ± 7.73	0.025

Abbreviation: BP, blood pressure.

^a^ Results are expressed as mean ± SD.

**Table 4.** Comparison of Heart Rate at Different Time Intervals Between the 2 Groups ^a^

Variables Name	Group 1	Group 2	P-Value
**HR_20 min**	80.39 ± 7.75	75.81 ± 10.53	0.039
**HR_1 h**	82.47 ± 14.69	74.53 ± 15.73	0.03
**HR_6 h**	97.36 ± 84.62	79.47 ± 10.75	0.212
**HR_9 h**	80.92 ± 6.61	77.14 ± 9.54	0.055
**HR_12 h**	80.89 ± 5.21	76.5 ± 7.96	0.007
**HR_18 h**	79.94 ± 7.01	76.19 ± 7.45	0.031
**HR_24 h**	78.58 ± 6.39	75.72 ± 7.92	0.096

Abbreviation: HR, heart rate.

^a^ Results are expressed as mean ± SD.

Mean total tramadol consumption (213.33 ± 44.08 vs 161.11 ± 37.93 mg, P-value 0.027; Table 5) was found to be significantly higher in group 1 than in group 2. Time to first rescue analgesia after TAP block in the postoperative period (47.5 ± 62.76 vs 77.22 ± 56.14 min, P-value 0.002) was significantly longer in group 2 than in group 1 (Table 5). Drug delivery attempts (19.14 ± 3.18 vs. 15.83 ± 2.81, P-value 0.001) and drug delivery counts (10.64 ± 2.19 vs 8.19 ± 2.14, P-value 0.001) through the PCA pump between groups (Table 5) showed a statistically significant difference.

**Table 5.** Comparison of Total Tramadol Consumption, Time to First Rescue Analgesia, and Drug Delivery Attempts and Drug Delivery Counts Through the PCA Pump Between the 2 Groups

Variables Name	Group 1	Group 2	P-Value
**Total Tramadol consumption**	213.33 ± 44.08	161.11 ± 37.93	0.027
**Time to first analgesia**	47.5 ± 62.76	77.22 ± 56.14	0.002
**Attempt**	19.14 ± 3.18	15.83 ± 2.81	0.001
**Delivered**	10.64 ± 2.19	8.19 ± 2.14	0.001

Abbreviation: PCA, patient-controlled anesthesia.

^a^ Results are expressed as mean ± SD.

In terms of overall satisfaction with pain management, 50% of group 1 and 61.1% of group 2 agreed on a Likert scale (Table 6), and a greater proportion of group 2 were satisfied with their overall pain treatment. In addition, 86.11% of group 1 and 69.4% of group 2 had no adverse effects. Nausea was seen in 11.1% of group 1 and 16.7% of group 2, sedation was observed in 2.8% of group 1 and 11.1% of group 2 (Table 7), and no significant side effects were found between the groups [P-value 0.279].

**Table 6.** Distribution of the Likert Scale for Overall Pain Management Between the 2 Groups ^a^

Variables Name	Frequency (%)		P-Value
	**Group 1**	**Group 2**	0.459
**Strongly disagree (1)**	0 (0)	0 (0)
**Disagree (2)**	3 (8.3)	2 (5.6)
**slightly disagree (3)**	8 (22.2)	5 (13.9)
**Slightly agree (4)**	7 (19.4)	5 (13.9)
**Agree (5)**	18 (50.0)	22 (61.1)
**Strongly agree (6)**	0 (0.0)	2 (5.6)
**Total**	36 (100.0)	36 (100.0)

^a^ Results are expressed as frequency and percentage.

**Table 7.** Frequency of Side Effects Observed in Both Groups ^a^

Side Effects	Frequency (%)		P-Value for Overall Side Effects Between the 2 Groups
	Group 1	Group 2	0.279
**No Side effect**	31 (86.11)	25 (69.4)
**Nausea**	4 (11.1)	6 (16.7)
**Sedation**	1 (2.8)	4 (11.1)
**Vomiting**	0	1 (2.8)
**Total**	36 (100)	36 (100)

^a^ Results are expressed as frequency and percentage.

## 5. Discussion

Postoperative pain after TAH is mainly due to somatic and visceral components, of which pain due to abdominal wall incision is more prominent (1). Transversus abdominis plane blocks are used in the treatment of acute postoperative pain after lower abdominal surgery and may be a better choice for pain control (2). The combination of spinal anesthesia and TAP block can provide effective pain relief after lower abdominal surgeries by minimizing the visceral component of pain as well (12). Hence, we preferred TAH under SAB in our study. The postoperative pain-free period is helpful for the patient in early ambulation, maintains a positive effect on mood and sleep, and decreases postoperative morbidity, hospital stay, and cost. Transversus abdominis plane block for lower abdominal surgery has been evaluated in many studies, comparing it with a placebo. The administration of a TAP block significantly reduced analgesic requirements (13).

The main results of our study reveal the superiority of dexmedetomidine as an adjuvant to levobupivacaine in TAP block in terms of a longer time to initial rescue analgesic administration, less tramadol consumption, and higher patient satisfaction with overall pain management. The mean VAS scores for the initial first hour were comparable between both groups, which could be due to the residual effect of SAB; however, afterward, at 6, 9, and 12 h, VAS scores were significantly lower in the dexmedetomidine group; this can be attributed to the fact that dexmedetomidine in combination with levobupivacaine prolonged the duration of analgesic action. Dexmedetomidine prolongs the duration of nerve block through vasoconstriction and inhibits the hyperpolarization-activated cationic current (14). A study was conducted using isobaric bupivacaine with 2 different adjuvants (dexmedetomidine or dexamethasone) in TAP block for postoperative analgesia in cesarean delivery. It was observed that the VAS pain score interpreted from patients decreased in the bupivacaine plus dexmedetomidine group (15).

Our study found that hemodynamic variables (mean BP and HR) were significantly different at some time intervals after the TAP block and that mean values of these hemodynamic parameters were lower in the dexmedetomidine group. This can be attributed to the anxiolytic and sympatholytic properties of dexmedetomidine (16).

Another study was conducted using ropivacaine with 2 different adjuvants (dexmedetomidine or dexamethasone) in TAP block for postoperative analgesia in cesarean delivery. They observed that the time to first rescue analgesia was longer in the ropivacaine plus dexmedetomidine group (17). Similarly, in our study, the requirement of first rescue analgesia after TAP block was later in the dexmedetomidine group and earlier in the dexamethasone group.

In our study, total tramadol consumption was higher in group 1 than in group 2, indicating the superiority of dexmedetomidine in relieving postoperative pain. We have also monitored drug delivery attempts and actual drug delivery counts by patients through the PCA pump among both groups, which can provide additional data to measure the quality of analgesia between groups. It was also observed that the mean number of attempts and actual delivery counts of drugs through the PCA pump was significantly higher in group 1 than in group 2.

Our study showed that patients receiving levobupivacaine with dexmedetomidine in the TAP block reported higher satisfaction scores for analgesia compared to patients receiving levobupivacaine with dexamethasone in the TAP block. However, when comparing both groups, we observed more instances of sedation in the dexmedetomidine group. Similarly, a study conducted by Thakur J et al. showed that the sedation score and patient satisfaction were higher in the group receiving bupivacaine with dexmedetomidine compared to the groups receiving bupivacaine alone and bupivacaine with dexamethasone (15). In our study, we used levobupivacaine instead of bupivacaine because it has a similar duration of action to bupivacaine but with fewer adverse effects (6).

Elhamamy compared bupivacaine, bupivacaine plus dexamethasone, and bupivacaine plus dexmedetomidine in the TAP block and found an increased duration of pain relief in the dexmedetomidine group, as indicated by VAS scores, which is consistent with our study. A longer time interval for the first rescue analgesia and decreased total analgesic consumption were also observed in the bupivacaine plus dexmedetomidine group (18).

Adverse effects such as sedation and postoperative nausea or vomiting were mild in our study. One patient in group 2 experienced vomiting, while nausea and sedation were more common in group 2 (dexmedetomidine) but were easily treated.

One limitation of our study is the absence of a control group (levobupivacaine without adjuvant) and its comparison with groups 1 and 2 to determine if it is worthwhile to add an adjuvant in the TAP block. Other limitations include inappropriate VAS scores in the first hour after surgery and the inability to compare the time of analgesic onset of action between groups due to the residual effect of SAB.

### 5.1. Conclusions

The addition of dexmedetomidine to levobupivacaine in the TAP block provides prolonged postoperative pain relief, reduced VAS pain scores, improved patient satisfaction, and decreased opioid requirements with fewer adverse effects compared to dexamethasone in patients undergoing TAH. However, the use of dexamethasone as an adjuvant remains a viable alternative, particularly due to its cost-effectiveness and lower incidence of adverse effects such as postoperative nausea and vomiting.

## Clinical Trial Registration Code:

CTRI/2021/01/030607.
